# Review of Micro/Nanotechnologies for Microbial Biosensors

**DOI:** 10.3389/fbioe.2015.00061

**Published:** 2015-05-11

**Authors:** Ji Won Lim, Dogyeong Ha, Jongwan Lee, Sung Kuk Lee, Taesung Kim

**Affiliations:** ^1^Department of Biomedical Engineering, Ulsan National Institute of Science and Technology, Ulsan, South Korea; ^2^Department of Mechanical Engineering, Ulsan National Institute of Science and Technology, Ulsan, South Korea; ^3^Department of Chemical Engineering, Ulsan National Institute of Science and Technology, Ulsan, South Korea

**Keywords:** micro/nanotechnology, microbial biosensor, microfluidics, bioreactor, micro/nanomaterials, sensory-regulative biosensor, riboswitch

## Abstract

A microbial biosensor is an analytical device with a biologically integrated transducer that generates a measurable signal indicating the analyte concentration. This method is ideally suited for the analysis of extracellular chemicals and the environment, and for metabolic sensory regulation. Although microbial biosensors show promise for application in various detection fields, some limitations still remain such as poor selectivity, low sensitivity, and impractical portability. To overcome such limitations, microbial biosensors have been integrated with many recently developed micro/nanotechnologies and applied to a wide range of detection purposes. This review article discusses micro/nanotechnologies that have been integrated with microbial biosensors and summarizes recent advances and the applications achieved through such novel integration. Future perspectives on the combination of micro/nanotechnologies and microbial biosensors will be discussed, and the necessary developments and improvements will be strategically deliberated.

## Introduction

**Figure 1:**
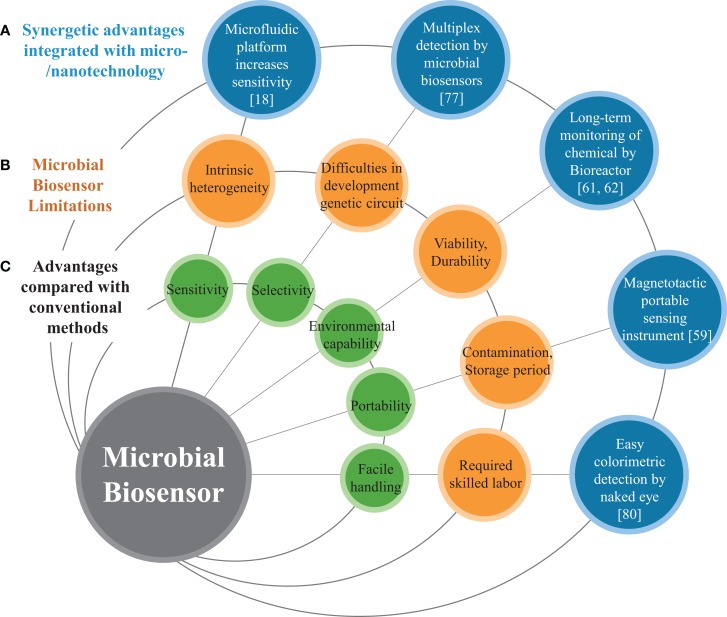
**Schematic diagram represents (A) micro/nanotechnolgies enhancing the performance of microbial biosensors, (B) limitations of conventional microbial biosensors, and (C) general features of biosensors**.

Biosensors are analytical tools that are generally used to detect or recognize specific elements. Since the first biosensor was developed by Clark in 1962 (Clark and Lyons, 1963), biosensors, with their great potential, have been widely studied and extensively applied in many situations. Typically, biosensors can be categorized by their fundamental platforms, including antibodies (Kusterbeck et al., 1990), protein receptors (Kricka et al., 2006), enzymes (Wilson and Hu, 2000; Ispas et al., 2012), and microorganisms (D’Souza, 2001; Lei et al., 2006; Su et al., 2011). Most of the biosensors in practical and clinical use in recent decades rely on enzymes (Mello and Kubota, 2002) and nucleic acid oligonucleotides with an array platform (Ehrenreich, 2006; Miller and Tang, 2009) due to their high specificity and sensitivity (Ispas et al., 2012). In parallel, as shown in Figure 1, microorganisms have been developed as biosensors and provide many advantages such as the ability to detect a wide range of substrates, reduced cost, mass production, and easier genetic modification compared to other platforms utilizing enzymes and mammalian cells (D’Souza, 2001; Lei et al., 2006; Su et al., 2011). However, determination of target compounds or environmental factors using microbial biosensors seems to be imprecise as it requires traditional analytical methods including test tubes or hand pipettes, making it highly dependent on the technical skill of researchers. In addition to such instrumental limitations, the relatively poor sensitivity and selectivity of microbial biosensors are still critical issues and this can be attributed to the nature of biological sensing mechanisms. Another intrinsic limitation of microbial biosensors is the slow response caused by decelerated diffusion of substrates and products through the cell wall (Su et al., 2011).

In this review, we discuss not only the academic applications of microbial biosensor development but also many cutting-edge micro/nanotechnologies developed for microbial biosensors. First of all, we discuss the basic principles of microbial biosensors. Second, we introduce conventional detection methods of microbial biosensors that are categorized by read-out method and have been widely adopted in many other review articles to date. Third, we review many recently developed micro/nanotechnologies, instruments, or miniaturization systems with automation functions that have been integrated with microbial biosensors. In particular, we focus on recent micro/nanotechnologies as a promising strategy to improve the sensitivity, selectivity, portability, and multiplexity of such microbial biosensors. We also review the advantages of the incorporation of micro/nanotechnologies into microbial biosensors over conventional methods to overcome the aforementioned limitations. Finally, we discuss recent biological technologies for enhancing the performance of microbial biosensors and several future perspectives.

## Microbial Biosensors

### Working mechanisms as biosensors

In recent decades, many improved microbial biosensors have been reported, which show promise for a wide variety of applications (Figure 1). Microbial biosensors are generally defined as analytical devices composed of a microorganism that detects a target substrate and converts the detected signal to a quantifiable response in a physiological, electrical, or biochemical manner. The sensing and recognition mechanisms of microbial biosensors include various types of conventional optical (e.g., fluorescence or bioluminescence) (D’Souza, 2001; Lei et al., 2006), electrochemical (Su et al., 2011), and sensory-regulated devices (Zhang and Keasling, 2011). Prior to delving into the conventional sensing mechanisms, we briefly review sensory-regulated genetic devices that are classified as emerging biotechnology for microbial sensing mechanisms.

Recently, novel molecular biological techniques have significantly improved microbial genetic manipulation and precise metabolic engineering for enhanced production of many useful biochemical signals. To regulate and optimize cell growth, behavior, and transduction of certain biochemicals, all biological systems have evolved with delicate sensory mechanisms. Sensory-regulative biosensors and their mechanisms can detect various cellular signals, and then transduce the signals in optical or electrochemical manner. Also, the regulation of cell behavior or metabolic pathways can be represented in other detectable manners because microorganisms detect not only environmental factors including nutrients, temperature, and pH but also sense their own metabolic status (Zhang and Keasling, 2011). In order to sense both intracellular and extracellular signals and then regulate the behavior of cell growth and responses, synthetically engineered biosensors including riboswitches (Dietrich et al., 2010), metabolite-responsive transcription factor-based biosensors, and other RNA biosensors (Winkler and Breaker, 2005) have been developed in genetic circuit forms by virtue of recent progress in synthetic biology. These newly developed microbial biosensors and their mechanisms provide an opportunity to sense other interesting metabolites/analytes with high sensitivity by allosterically regulating the metabolic pathways of microorganisms. The details of biotechnological approaches to microbial biosensors seem to be beyond the scope of this review.

### Advantages and limitations

As mentioned earlier, microorganisms such as bacteria and yeast offer a promising strategy for developing microbial biosensors that possess various advantages from many perspectives. First, biosensors based on microorganisms offer an analysis cost for sensing elements that is considerably lower than that of other methods requiring conventional instruments such as gas chromatography, liquid chromatography, mass spectrophotometry, and other methods (Arefev et al., 1987). Since microorganisms can be produced in massive numbers using a simple culture process and cheap liquid nutrient media, the analysis cost can be dramatically reduced. Second, microbial biosensors show the potential to detect various target elements, and the engineering of such microbial biosensors for specific substrates appears to be easily achieved by using recent molecular biological techniques (Zhang and Keasling, 2011). Genetic manipulation of microorganisms seems to be better controlled and tailored than engineering mammalian, plant, or other types of biosensors (Mulchandani and Rogers, 1998). Third, other types of representative biosensors such as those based on enzymes or antibodies are comparably unstable and very sensitive to pH and temperature. However, microbial biosensors that can be more robust show an excellent capacity to endure various environmental conditions. Despite the multiple advantages of microbial biosensor over conventional sensing instruments, the widespread use of microbial biosensors is hampered by a few intrinsic limitations of microorganisms such as comparably low sensitivity, which is closely coupled with both cell population size and optical signal (Casavant et al., 2003; Kim et al., 2014), poor selectivity in multiplex detection (Jouanneau et al., 2011), intrinsic cellular heterogeneity both genotypically and phenotypically (Brehm-Stecher and Johnson, 2004), and stochastic protein expression (Swain et al., 2002).

## Conventional Detection Methods

### Optical detection methods

Optics has played an important role in biosensor development as a fundamental tool for sensing signals. Microbial biosensors that detect interactions between microorganisms and analytes are no exception. Such interaction induces an engineered genetic circuit in a microorganism to activate a reporter gene for expression of a measurable signal. To quantify the optical signal, which is sensitive enough to figure out the interaction between the reporter and inducer molecules, diverse detection systems have been developed. In the early days of development, a photon detector, which absorbs photons using a semiconductor film to form electrons and holes for creating a current, was used to detect luminescent signals in response to pathogens (Shaw and Kado, 1986). Fluorescence microscopy is also used for its wide range of applicability, which can not only measure signal but also provide *in situ* imaging (Joyner and Lindow, 2000). Chromatography techniques such as high-pressure liquid chromatography are very simple tools that are widely used for detecting colorimetric signals, because the signal can be even observed with the naked eye (Fujimoto et al., 2006; Santos and Stephanopoulos, 2008; Di Gennaro P et al., 2011; Rocaboy-Faquet et al., 2014). Following the introduction of microwell plates, use of conventional optical technologies in microbial biosensors became popular, and has led to the use of microbial biosensors in many applications. In particular, microwell plates have been successfully integrated with luminometers, which measure the intensity of luminescent light, to estimate adenosine triphosphate or luciferase and then used for most luminescence-based biosensor experiments (Bontidean et al., 1999; Petanen and Romantschuk, 2002; Kim and Gu, 2003). Also, fluorescence spectrometers, which are composed of a diffraction grating structure to make a light source monochromatic and a photomultiplier tube to quantify the fluorescent light, are used for fluorescence-based experiments (Taylor et al., 2004; Wells et al., 2005; Keenan et al., 2007).

### Electrochemical detection methods

Electrochemical microbial biosensors are one of the most widely used platforms for microbial biosensors because of their high-sensing accuracy (D’Souza, 2001) and possible applications such as point-of-care testing devices (DeBusschere and Kovacs, 2001). Therefore, many researchers and industries have introduced electrochemical microbial biosensors that can detect many types of target materials such as glucose (Kohlmeier et al., 2008; Odaci et al., 2008a), heavy metal ions (Chouteau et al., 2005; Guedri and Durrieu, 2008), phenol (Kirgöz et al., 2006; Neufeld et al., 2006), and other chemicals (Mulchandani et al., 2001; Tkac et al., 2003; Lei et al., 2006; Tag et al., 2007). Electrochemical microbial biosensors generally consist of a working electrode, a transducer layer for detection (microorganisms), and recording equipment. The signal from the transducers, produced by the electrochemical reaction, is recorded and correlated with the concentration and composition of the chemical compounds present, and displayed as an electrical expression. These systems can be classified according to the mechanism used to detect the signal from the transducer: (1) conductometric-, (2) amperometric-, (3) potentiometric-, and (4) voltammetric microbial biosensors (Su et al., 2011).

Conductometric microbial biosensors detect chemicals by the variation in conductivity of a sample solution via the consumption or production of ions by transducers. They can rapidly detect target chemicals with high sensitivity. In particular, they can easily be miniaturized because they do not require a reference electrode (Shul’ga et al., 1994). However, they have a low selectivity for chemical compounds because the variation in conductivity can be affected by electrical charges (Mikkelsen and Rechnitz, 1989). Amperometric microbial biosensors express the chemical concentration by recording the current signal through a sample (Ding et al., 2008). In particular, amperometric microbial biosensors can provide outstanding sensitivity, owing to the advances made in the current measuring device (<pA) (Su et al., 2011). Potentiometric approaches use the potential difference from a reference (or grounded) electrode, and thus require three electrodes, two working electrodes and a reference electrode. Two major advantages of potentiometric electrochemical microbial sensors are their selectivity for target chemicals and their remarkable sensitivity. However, they are limited by their requirement for a reference electrode for stable and accurate sensing (Su et al., 2011). Voltammetric microbial biosensors are a comparably versatile platform for the detection of chemical compounds; they record and correlate each electric signal (electric current and potential difference) with a corresponding sample (Wang and Wang, 1985). Voltammetric approaches can provide high selectivity and measurability via the position and density of the peak current signal. However, they require complex components and their detection speed is low.

Currently, micro/nanotechnologies are being rapidly applied to and integrated with electrochemical detection technologies that employ microbial biosensors (Durrieu et al., 2013; Gokhale et al., 2013). The principal goals of such integration of micro/nanotechnologies with electrochemical microbial biosensors are for (1) miniaturization and portability, (2) high-throughput screening, (3) enhanced sensitivity and selectivity, and (4) simple and rapid immobilization of microorganisms (transducers), which replaces conventional transducers (Scognamiglio, 2013).

### Detection equipment

Conventional detection equipment for microbial biosensor like microplate readers has been used for establishing the fundamental methods for selecting superior microorganisms, detecting toxic compounds, or monitoring environmental conditions (Petanen and Romantschuk, 2002; Kim and Gu, 2003; Santos and Stephanopoulos, 2008). However, such microplate readers could not completely fulfill the requirement of microbial biosensors. In fact, not only do they show weaknesses in throughput, portability, sensitivity, and selectivity but they also still require the skilled labor to implement the biosensing process. Although microwell plate-based detection has proven useful for enhancing throughput and reducing the consumption of resources, these endeavors did not overcome the limitations of even higher sensitivity, full automation, or provide a portable usage environment. These unsolved complications became the motivation for novel integration with micro/nanotechnology, which is attracting attention from both the scientific and industrial communities.

## Micro/Nanotechnological Detection Methods

To overcome the limitations of conventional detection methods, several examples of innovative integration of microbial biosensors with recent micro/nanotechnologies have been proposed in the past decade (Table 1). For instance, microfluidic systems showed many advantages by minimizing the sample and reagent volumes required, shortening analysis time with high resolution and repeatability, and demonstrating multiple assays on a chip in a high-throughput manner (Kim et al., 2014). In addition, it was demonstrated that microfluidic systems can not only provide microorganisms with an ideal cell-culture microenvironment that is close to *in vivo* one (Shaw and Kado, 1986) but also enable high portability and more rapid analysis compared to conventional methods (Joyner and Lindow, 2000). Moreover, micro/nanofabrication showed remarkable potential for microbial biosensors from the viewpoint of (1) enhanced optical and electrochemical measurements, (2) improved immobilization and automated culture environments, and (3) high portability and more practical applications. In the following sections, various micro/nanotechnologies that can effectively improve microbial biosensors will be discussed in comparison with conventional analytical methods (Fujimoto et al., 2006).

**Table 1:** **Comparison of microbial biosensors integrated with micro/nanotechnologies**.

Integrated technology	Microorganism	Detection method (transducer)	Substrate	Dynamic range/(LOD)	Improvements
**Instrument**
Automated	*S. cerevisiae*	Fluorescence	Methylmethan sulfonate (MMS)	0.01%	Reducing time compared with Ames Test (6 times faster) (Knight et al., 1999)
Automated	*J. lividum*	Luminescence	EC_50_		Real-time automated toxicity monitoring for a month (Cho et al., 2004)
Portable	*E. coli*	Luminescence	Phenol	0.15 ~ 5 mM	Increasing the retention period by using freeze-drying method (Choi and Gu, 2002)
			Mitomycin C	0.27 ~ 2 ppm	
			H_2_O_2_	0.0006 ~ 0.0025%	
			Ethanol	1 ~ 3%	
Portable	*E. coli*	Luminescence	Benzene	0.5 ppm	Introduction of battery for *in situ* test (Berno et al., 2004)
Multiplexed	*E. coli*	Luminescence	Arsenic	5 μM	Multiplexed detection by immobilization in multi-well kit (Charrier et al., 2011)
			Cd	0.5 μM	
**Microfluidics**
Microwell	*E. coli*	Fluorescence	Hg	100 nM	Improved sensitivity by separating the *E. coli* individually (Biran et al., 2003)
Compact disk	*E. coli*	Fluorescence	Arsenite	1 μM ~ 5 mM	Reducing the consumption of resource by miniaturized platform (Rothert et al., 2005)
			Antimonite	
PDMS chip	*S. cerevisiae*	Fluorescence	MMS		Reducing the consumption of resources by miniaturized parallel detection system (García-Alonso et al., 2009)
PDMS chip (magnetic)	*S. cerevisiae*	Fluorescence	MMS	0.28 μM ~ 450 μM	Improved sensitivity by regulating the position of yeast (García-Alonso et al., 2011)
PDMS chip (magnetic)	*M. gryphiswaldense*	Luminescence	Dimethyl sulfoxide (DMSO)	2 ~ 50%	Improved sensitivity by regulating the position of *E. coli* (Roda et al., 2013)
			Taurochenodeoxycholic acid (TCDCA)	0.001 ~ 10 mM	
PDMS chip	*E. coli*	Fluorescence	Cd	2 nM ~ 20 μM	Improved sensitivity by accumulating *E. coli* and multiplexed design (Kim et al., 2014)
			Hg	2 nM ~ 20 μM	
Microfluidic	*G. sulfurreducens*	Amperometric	Anthraquinone disulfide (AQDS)		Live monitoring, quantitative analysis (Li et al., 2012)
**Bioreactor**
Miniaturized bioreactor	*E. coli*	Luminescence	Ethanol	3.4%	Reducing time and the consumption of resources by miniaturized bioreactor; (Gu et al., 1996)
Miniaturized bioreactor	*E. coli*	Luminescence	Tributyltin	0.02 μM	Improved sensitivity by regulating the oxygen and pH (Thouand et al., 2003)
Miniaturized bioreactor	*E. coli*	Luminescence	Pheonl	300 ppm	Multiplexed detection by miniaturized parallel bioreactor (Gu and Gil, 2001)
			Mitomycin C	50 ppb	
			Cerulenin	5 ppm	
**Micro-/nanofabrication**
Photolithography	*E. coli*	Voltametric	*p*-aminophenol		Miniaturization, eight testing chamber on single chip (Popovtzer et al., 2005)
Screen printing	*E. coli*	Amperometric	Methyl parathion	2 ~ 400 μM	Miniaturization, reproducibility, stability (Shitanda et al., 2009)
DRIE process	*E. coli*	Amperometric	Nalidixic acid		Improved detection signal (Ben-Yoav et al., 2012)
**Micro-/nanomaterials**
Carbon nanotube (CNT)	*P. putida*	Amperometric	Phenol	0.5 ~ 4 mM	Prevent electric noise signal (Timur et al., 2007; Odaci et al., 2009)
Microfiber-nanoparticle	*E. coli*	Amperometric	Glucose	0.25 ~ 0.55 mM	Self-assembly of nanoparticle (microfiber), improved electric properties (Deng et al., 2010)

### Microfluidics

Microfluidic technology has been used for a broad range of biological assays. Especially microfluidic technology provides various, miniaturized cell-culture environments in a small scale, which facilitate not only sensitive (Park et al., 2012; Kim et al., 2014) and parallel analysis (Si et al., 2012) of cell cultivation and/or fermentation in a high-throughput manner but also the concentration gradient generation for multiplex analysis (Park et al., 2011; Kim et al., 2014). It is noted that this feature is appropriate for the microbial biosensor because microfluidics technology reduces cost and labor and improves sensitivity and selectivity with high resolution. For example, Biran et al. (2003) fabricated a novel microwell by using optical fiber to improve the sensitivity. A different etching rate between the core and the cladding in the optical fiber was used to form a well on the core part and a wall on the cladding part. Since the core size was appropriate for immobilizing a single cell in each microwell, it facilitated multiple individual microbial biosensors with high sensitivity. Using this method, the mercury concentration was able to be detected by measuring the fluorescent signal from individually immobilized *Escherichia coli* RBE27-13 harboring pECFP. Also, Rothert et al. (2005) reported a centrifugal microfluidic platform integrated with the microbial biosensor to reduce time and resource consumption, and increase portability. Computerized numeric control machining was used to fabricate poly(methyl methacrylate) in the shape of a compact disk, and centrifugal forces made the mixing process efficient between the reagent and *E. coli* AW10 harboring pSD10. This microfluidic platform reduced the resources consumed but the analytical performance of the microbial biosensor for detecting arsenite and antimonite was not affected, showing the advantage of microfluidic integration with microbial biosensor.

Additional miniaturization was incorporated by using micro/nanofabrication technologies and was further facilitated by soft-lithography (Xia and Whitesides, 1998). In particular, microfluidic devices are made of polydimethylsiloxane (PDMS), which is a representative material for microfluidics, is transparent and biocompatible, and is appropriate for a biosensor platform. Because soft-lithography allows flexible channel design, microfluidic devices can be used for microbial biosensors to screen different toxic compounds in separated and parallel channels, enabling a high-throughput assay on a chip. For instance, García-Alonso et al. (2009) reported eight parallel microfluidic channels used for detecting methyl-methanesulfonate, depending on its concentration. This tool facilitates a rapid qualitative measurement of the harmfulness of the toxic material on the *Saccharomyces cerevisiae* RAD54.

**Figure 2:**
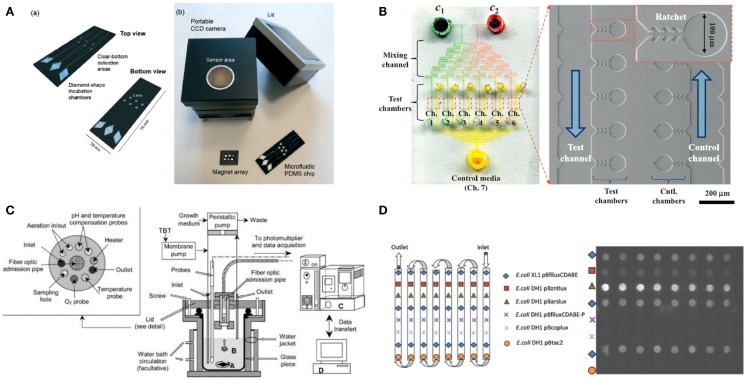
**Various micro/nanotechnologies for enhancing the performance of microbial biosensors**. **(A)** A magnetotacic array device was introduced that can improve the positioning of microbial biosensor by separating a detection area from a cultivation area. The figure is reprinted with the permission from Roda et al. (2013) [Copyright (2013) Royal Society of Chemistry]. **(B)** A microfluidic device was developed for multiplex detection of small volume samples (Kim et al., 2014). The image is reproduced with the permission from Kim et al. [Copyright (2015) Elsevier B. V.]. **(C)** Miniaturized bioreactor facilitates not only the cultivation of bacterial cells but also the real-time monitoring of toxic material at a practical level. The image is reprinted with the permission from Thouand et al. (2003) [Copyright (2003) Springer International Publishing AG]. **(D)** A removable multi-well card was introduced for automated detection of multiple components. The image is reprinted with the permission from Charrier et al. (2011) [Copyright (2003) Springer International Publishing AG].

A PDMS microfluidic device for a microbial biosensor has been developed to improve sensitivity by magnetically controlling position or increasing the number of bacterial cells. For example, a microfluidic device integrated with magnetically functionalized reagents was developed by García-Alonso et al. (2011), which facilitated removal and relocation of microbial biosensors conveniently. The yeast cell was magnetized for easy handling of its position by coating polyallylamine hydrochloride (PAH)-stabilized magnetic particles, which are positively charged (ca. 15 nm in diameter). Since the used cells can be easily discarded by the flow of culture media without external magnets, the device can be reused with good reproducibility. The technique appears to resolve the problem of cell retention, which is a major hurdle in the development of microfluidic devices, and make it easy to control the nutrient conditions and analyte input. Also, a novel magnetotactic bacterium, *Magnetospirillum gryphiswaldense* MSR-1, has been developed by Roda et al. (2013) (Figure 2A). A permanent magnet trapped the bacteria in a detection area that was in contact with a charge coupled device sensor. Since the position of the bacteria could easily be controlled, not only it was easy to wash them out and reuse the instrument but it also increased sensitivity by decreasing the noise-to-signal ratio because the culturing and detecting positions were placed far apart. In addition, a novel microfluidic device was introduced that allowed continuous supply of nutrient for increasing cell growth rates and the number density in a microchamber (Figure 2B) (Kim et al., 2014). Because the device implemented a microfabricated ratchet structure to prevent motile bacterial cells, *E. coli* HK621 and HK622, from escaping from a culture microchamber, the accumulated fluorescent signals were significantly amplified over time. The microfluidic device increased the sensitivity of microbial biosensors over three orders of magnitude compared to conventional methods in heavy metal detection. In addition, it showed high potential for a high-selective microbial biosensor platform.

### Microbioreactors

For various biological assays, optimization studies for initial environmental conditions are typically performed in miniaturized-scale under several conditions as similar as possible to the actual large-scale conditions for industrial cultivation and fermentation. These optimization approaches are often called the scale-down approach (Kumar et al., 2004). Gu et al. (1996) developed a miniaturized bioreactor for reducing the working volume while retaining the main function of the conventional bioreactor. This bioreactor was composed of a culturing chamber, injected air, a water-based temperature controller, cell inoculation, and chemical injection parts. This instrument made it possible to conduct long-term continuous experiments using only a small amount of medium; it required approximately 4 L/week by using *E. coli* TV1061 harboring pGrpELux5. Based on the initial miniaturized bioreactor mentioned earlier, a multi-channel bioreactor has also been developed for detecting multiple components (Gu and Gil, 2001). Four different bioluminescent bacteria, *E. coli* DPD2794, DPD2540, TV1061, and GC2, were placed in each channel for testing, for example, water samples. Since the small size allowed minimal media consumption and easy setup, this instrument was very economical. Thouand et al. (2003) have improved a bioreactor through the addition of oxygen and a pH controller (Figure 2C). These additions allowed more sensitive detection due to stricter regulation, which is basically important for cell growth rates.

### Micro/nanofabrication

Micro/nanofabrication processes have been actively developed and applied to various research and industrial fields during the last two decades. This seems to be possible because of numerous developments in machining tools and measuring equipment (Chen and Pepin, 2001). In this context, micro/nanofabrication techniques are also applied to and integrated with electrochemical detection using microbial biosensors. Typically, micro/nanofabrication is combined with microbial biosensors for several improvements such as a stable and simple process for transducer immobilization and miniaturization for high-throughput screening (Scognamiglio, 2013).

**Figure 3:**
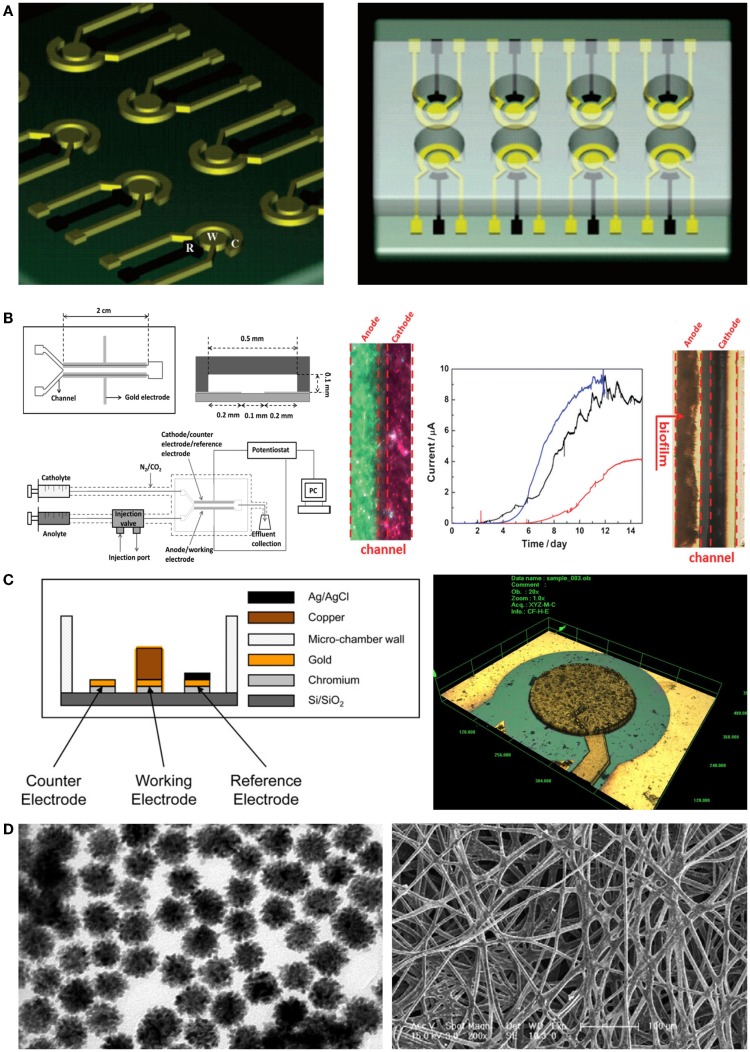
**Novel micro/nanoscale structures and materials for enhancing the performance of electrochemical detection of microbial biosensors**. **(A)** A miniaturized microbial biosensor was integrated with eight electrochemical sensing cells fabricated by photolithography techniques. Toxic materials such as phenol and ethanol in water were detected in a high-throughput manner. The figure is reproduced with the permission from Popovtzer et al. (2005) [Copyright (2005) American Chemical Society]. **(B)** A microfluidic device enabled microbial biosensors to conduct quantitative analysis and live monitoring of AQDS. Laminar flows generated by a Y-shape microfluidic channel network made it possible to reduce reaction and response time in electrochemical detection. The figure is reproduced with the permission from Li et al. (2012) [Copyright (2012) John Wiley and Sons, Inc.]. **(C)** Electrodes were fabricated by using microfabrication techniques including deep reactive ion etching and then applied to microbial biosensors. Since the microstructured electrodes enhanced electric signal from microbial biosensors, the induction factor improved over two times. The figure is reproduced with the permission from Ben-Yoav et al. (2012) [Copyright (2012) Elsevier B. V.]. **(D)** Metallic nanoparticles integrated with silk microfibers showed remarkable sensing ability for the detection of glucose in various concentrations. The figure is reproduced with the permission from Deng et al. (2010) [Copyright (2010) Elsevier B. V.].

The photolithography technique is a fundamental micro/nanofabrication process for fabrication of miniaturization systems. Micropatterned electrodes can easily be fabricated on a silicon wafer using a photo mask and vacuum evaporation of metallic ions such as gold and platinum (Claussen et al., 2009). Therefore, a miniaturized electrode can dramatically increase the reaction speed of sensors when applied to electrochemical microbial biosensors. For example, Popovtzer et al. (2005) suggested using photolithography for detection of water toxicity by microbial biosensors (Figure 3A). Eight miniaturized sensor cells were integrated on a single disposable chip with a partial gold coating, allowing individual operation. Each chamber consisted of three embedded electrodes: a gold working electrode, a counter electrode, and a reference electrode. Using the fabricated chip, they measured the potentiostatic signal from microbial biosensors and then determined the presence of ethanol and phenol in water. In addition, with recent advances in photolithography techniques, many microfluidic devices have also been combined with electrochemical microbial biosensors. The integration of microfluidic devices provides numerous advantages for high-throughput screening via miniaturization. Li et al. (2012) reported a laminar flow-based microfluidic device for live monitoring and quantitative analysis of anthraquinone disulfide (AQDS) in solution (Figure 3B). In particular, they used laminar flow in microchannels for elimination of the separation membrane, which was an essential element in previous microfluidic devices. Furthermore, it can provide a short hydraulic retention time (ca. 2 min) and a rapid response time (<21 min) for *Geobacter sulfurreducens* cells by continuous provision of substrate.

When combined with electrochemical microbial biosensors, screen-printed electrodes (SPEs) provided several improvements such as a low detection limit, a simple fabrication process, and a wide range of printing materials. Additionally, SPEs were used for enhanced immobilization of microorganisms on the working electrode (Alonso-Lomillo et al., 2010). Shitanda et al. (2009) fabricated an amperometric microbial biosensor using a carbon electrode on which biomaterials had been printed. The ink suspension (algae, sodium alginate solution, and cells) was printed onto the screen-printed carbon electrode and directly immobilized there via cross-linking using a CaCl_2_ solution. Hence, the microbial biosensor could amperometrically detect atrazine, using *Chlorella vulgaris* cells. This device introduced a simple immobilization process and demonstrated cost-effectiveness and high portability compared with previous algal biosensors. Another miniaturized electrochemical microbial biosensor was developed by Ben-Yoav et al. (2012) (Figure 3C). They integrated a pillar structure on a silicon wafer and coated a metallic substrate with three-dimensional (3D) nanostructures, using the deep reactive ion etching process, electrodeposition, and electro-polymerization of a conducting polymer (polypyrrole, PPy). They confirmed the effects of electrode materials such as copper and gold. Additionally, they investigated the effect of increasing the surface area using an electrodeposited and PPy-coated 3D micro/nanostructure. Finally, they successfully showed that surface-modified electrodes can significantly increase the signal from microorganisms.

### Micro/nanomaterials

Micro/nanomaterials have been drawing significant attention for electrical and chemical modification of substrates because they possess outstanding electrochemical properties derived from the large surface-to-volume ratio and the rapid transport of electrons. For this reason, many researchers attempted to employ micro/nanomaterials to modify and/or functionalize electrodes and then integrate them with microbial biosensors. As a result, the sensitivity of electrochemical microbial biosensors significantly improved. The most popular micro/nanomaterial seems to be carbon nanotubes (CNTs) (Odaci et al., 2008b) because addition of CNTs can easily modify electrodes. For example, they can increase electrical conductivity, functionalize cationic surfactant to stabilize certain molecules, and improve response time toward, thereby resulting in the improvement of microbial biosensors. Of course, conventional CNT-based electrodes have some weaknesses such as high-background current (noise) and a decreased electron diffusion rate during operation due to overlapping of the diffusion layer. Timur et al. (2007) introduced a method for modifying CNT-based electrodes using a mixture of CNT and redox osmium (Os-redox) polymer solution to overcome the noise-to-signal ratio limitations (Odaci et al., 2009). They optimized the required conditions for phenol detection using *Pseudomonas putida* cells. This was possible by using a mixture of CNT and Os-redox polymer and manipulating pH and temperature as well.

In addition, some different and integrative approaches were taken for modifying electrodes with nanoparticles and microfibers. In particular, Deng et al. (2010) developed a novel device for electrochemical microbial biosensors by using a silk-derived (S-derived) carbon fibrous mat with metallic nanoparticles (Au–Pt) (Figure 3D). The micro/nanomaterial used in their work contained amino groups in the fibrous component, allowing self-assembly of nanoparticles on the carbon fibrous mat. The immobilization of S-derived carbon fibers on nanoparticles allowed the efficient electron tunneling that in turn amplified the electrical communication between the microbial biosensor and the electrode surface. This resulted in a novel microenvironment that sustained the bioactivity of microbial biosensors, showing high sensitivity and a low detection limit compared with commercialized carbon paper-based biosensors.

## Micro/Nanotechnological Platforms for Microbial Biosensors

### Automation, portability, and multiplexity platforms

Micro/nanotechnology has contributed to the development of new instruments for (1) fully automated processes (Cho et al., 2004), (2) miniaturization for portability (Choi and Gu, 2002), and (3) complexed multicomponent detection (Charrier et al., 2011). First, a semi-automated system has been developed by Knight et al. (1999) to reduce the time required for measuring the fluorescent signal of RAD54 protein in *S. cerevisiae*. The combination of a laser light source, a detector, and an automated cell-culture chamber enabled continuous measurements of fluorescent signals, which in turn were rapidly processed in real-time. Although it was a prototypic semi-automated instrument, a reliable result could be acquired six times as fast as using a standard colony-based growth test. An instrument has also been developed by Cho et al. (2004) for automated and continuous detection systems with low cost, which was composed of robot arms, multiple microwell plates, a temperature controller, and a photomultiplier tube sensor used for measuring the intensity of light with a photoelectron. The robot arms made it possible to conduct experiments continuously, without manual control, enabling real-time toxicity monitoring at 10-min intervals for up to a month. Using this instrument, the toxicity of wastewater samples without further purification was detected by *Janthinobacterium lividum* YH9.

Second, significant research efforts have been made for portable detectors because portability became an important issue for practical applications of microbial biosensors and the demand for *in situ* testing had led to the improvement of portable microbial biosensors. Most recognition and detection systems are composed of a biosensing chamber, a light-proof chamber, and a luminometer. For example, a freeze-drying method was developed by Choi and Gu (2002) to extend the period of use of the portable biosensor so that sensor cells, *E. coli* DPD2794, DPD2540, TV1061, and GC2, can easily be transported and used for environmental detection and monitoring after rehydration of lyophilized cell. This portable biosensor kit showed remarkable potential for practical application; it was achieved by increasing the retention period. Also, a portable microbial biosensor was reported by Berno et al. (2004), which detects benzene not only in laboratory samples but also in outdoor samples for *in situ* testing. *E. coli* HB101 harboring pTSN316 cultured on solid-soil dish could be used in outdoor due to portable colorimeter integrating with batteries and alimentation support.

Third, basically the multiplexity of a biosensor determines practicality because the performance of the biosensor is decided not only by its portability but also by its capability to deal with multiple components in a single process. For example, Charrier et al. (2011) reported that integration of removable multi-well cards, an optical setup for bioluminescence monitoring, a fluidic channel network for media and sample loading, and a computer interface for full automation, allowed the detection of multiple components (Figure 2D). Four bacterial cells, *E. coli* DH1 pBzntlux, pBarslux, pBcoplux, and XL1 pBfiluxCDABE, were immobilized in an agarose matrix on a multi-well card, media and samples were flown along the fluidic channels, bioluminescent signals from *E. coli* were measured by a CCD camera, and all experiments were controlled and all data processing were automated by a computer. Although the microbial biosensors showed intrinsic weaknesses in cross-talk and synergistic effects for a heavy metal mixture, it was well demonstrated that a multiplex detection using microbial biosensors can be incorporated. In addition, Struss et al. (2010) reported a simple and portable microbial biosensor that employed filtering paper strips and a colorimetric detection method. A chromogenic substrate was functionalized to express a visible signal through an interaction between *N*-acylhomoserine lactones (AHLs) and β-galactosidase. Since AHLs that are known as quorum sensing molecules accelerate the formation of analyte-induced optical signals it was well demonstrated that microbial biosensors integrated with the filtering paper strips were used for a simple, portable biosensor. Potentially, the method appears to provide multiplex detection methods using microbial biosensors because the filtering paper strips can be easily amended independently and separately, facilitating multiplex detection on a run.

### Screening platform

Up to now, we reviewed that a broad range of micro/nanotechnlogies is suitable for microbial biosensors to better detect target chemical compounds and/or environmental factors. In this section, we shortly discuss that micro/nanotechnologies have high potential for providing an unprecedented screening platform that overcomes critical technological limitations of conventional screening platforms including instruments and methods. In particular, it is worthy to discuss micro/nanotechnological screening platforms for microbial biosensors because they facilitate the screening process of a large number of combinatorial library in a high-throughput manner; the larger is the mutant library size, the higher the chances are expected to find desired, optimal microbial biosensors (strains). Of course, the sensory-regulative biosensors are closely correlated to various screening platforms for the identification of synthetic biosensors. On the other hands, the conventional screening platforms appear to be unsuitable for dealing with such a large mutant library size because of the low throughput (Zhang and Keasling, 2011).

Here, we reviewed two representative micro/nanothnological screening platforms. First, Baret et al. (2009) suggested a highly efficient microfluidic droplet sorting platform that enabled the high-throughput analysis of microbial enzyme activity. Microorganisms were engineered to express intracellular enzyme β-galactosidase and then their mutant libraries were produced. To screen out the mutant libraries, they were encapsulated and compartmentalized in individual droplet. According to the presence or absence of microbial enzymatic activity, the microfluidic platform made it possible to sort target microorganisms of the mutant libraries. This platform demonstrated that micro/nanotechnologies can facilitate high-throughput screening of microbial biosensors as well. In addition, Wang et al. (2014) successfully integrated a microfluidic platform with a sensory-regulative microbial biosensor for cellular metabolite analysis. The platform allowed the high-throughput analysis of extracellular, secreted metabolites and recognition of genetic elements that were responsive to allosteric regulative effects. Using the microfluidic platform with sensory-regulative riboswitches, the xylose over-consuming strain was effectively enriched and identified as a representative result. It appears to be impossible to achieve such accomplishment using conventional experimental platforms, especially due to the limited throughput. In other words, it is obvious that not only the selection of extraordinary sample but also the identification of riboswitches benefited from micro/nanotechnologies.

Although these types of integrative approaches have just begun the very first step toward the high-throughput screening platform, it is highly believed that micro/nanotechnologies can effectively incorporate a novel screening platform for microbial biosensor that can be further accelerated with the aid of sensory-regulative biosensors and riboswitches.

## Conclusion and Future Perspectives

In this review, we have discussed the integration of micro/nanotechnologies with microbial biosensors and their applications. Microbial biosensors have been under a wide range of investigations in recent decades, but they seem to be typically limited by several factors such as low sensitivity, poor selectivity, difficult sensor engineering, and stochastic heterogeneity. With the rapid expansion of interdisciplinary convergence research, microbial biosensors have been integrated with many recent micro/nanotechnologies to overcome such limitations.

First of all, micro/nanotechnologies have contributed to improving the performance of optical microbial biosensors by considerably ameliorating the problems posed by conventional optical detection methods. In parallel, micro/nanotechnologies have revolutionalized the electrochemical detection sensitivity and selectivity of microbial biosensors. To date, many attempts made to combine micro/nanotechnologies with microbial biosensors were proven successful because they fulfilled various demands from the industrial and environmental fields through miniaturization and high-throughput assay on a run. In addition, automation and miniaturization of instruments and bioreactors with optical/electrochemical detection systems allowed microbial biosensors to be used in an effective, efficient, and practical manner. Development of portable detectors and supporting tools has raised the possibility of previously impractical applications. Furthermore, micro/nanofluidics further incorporated real miniaturization of the culture environment for microbial biosensors, reducing the consumption of resources and increasing their sensitivity from the viewpoint of optical detection. Automated and miniaturized systems for multiplex detection suggested new analytical methods for the identification of real, unknown multi-samples with improved selectivity. In particular, many microbial biosensors integrated with micro/nanomaterials showed many unique advantages including high sensitivity, high selectivity, and rapid response time with high resolution and accuracy.

Also, sensory-regulative biosensors are emerging as a novel sensing mechanism. These innovative regulative microbial biosensors, including riboswitches, require efficient screening methods to select extraordinary samples from numerous possible combinations. Hence, micro/nanotechnological screening platforms such as microfluidics platforms have been introduced with an effective integration strategy. Far more practical platforms than presented here will be developed and used to provide wider insight into how the microbial pathway dynamically controls the overall microbial status, including cell viability, genetic communication processes, and up–down regulation of productivity for various targets.

Nevertheless, many hurdles in micro/nanotechnology-assisted microbial biosensors still remain before they will successfully substitute for other types of artifactual biosensors. Although microbial biosensors with high portability and high multiplexity have been widely studied and even demonstrated, several challenging issues should be taken into serious consideration and be fully addressed. The issues requiring further investigation are immobilization technique, late response time, intrinsic toxicity of chemicals, solvents, and micro/nanomaterials, bio-compatibility of fabrication processes and materials, long-term cultivation and reusability, and contamination and shelf-life of microbial biosensors. For instance, unless a long shelf-life without unwanted contamination is guaranteed, microbial biosensors cannot substitute for other similar biosensors because any contamination of culture media or other sources may nullify the function of the microbial biosensors. Lastly, we believe that not only will advanced micro/nanotechnologies herald the beginning of a new era for microbial biosensors but also many interesting possibilities and promising opportunities within the field of microbial biosensors still remain. Therefore, successful and strategic integration between microbiological sciences and micro/nanotechnologies will thus unlock the full potential of microbial biosensor technology in the near future.

## Conflict of Interest Statement

The authors declare that the research was conducted in the absence of any commercial or financial relationships that could be construed as a potential conflict of interest.
